# Sotagliflozin Added to Optimized Insulin Therapy Leads to Lower Rates of Clinically Relevant Hypoglycemic Events at Any HbA1c at 52 Weeks in Adults with Type 1 Diabetes

**DOI:** 10.1089/dia.2019.0157

**Published:** 2019-08-19

**Authors:** Thomas Danne, Jeremy Pettus, Andrea Giaccari, Bertrand Cariou, Helena Rodbard, Stuart A. Weinzimer, Mireille Bonnemaire, Sangeeta Sawhney, John Stewart, Stella Wang, Rita de Cassia Castro, Satish K. Garg

**Affiliations:** ^1^ Diabetes Center, Children and Youth Hospital Auf der Bult, Hannover Medical School, Hannover, Germany.; ^2^ Department of Medicine, University of California San Diego, San Diego, California.; ^3^ Center for Endocrine and Metabolic Diseases, Fondazione Policlinico Universitario A. Gemelli IRCSS, Università Cattolica del Sacro Cuore, Rome, Italy.; ^4^ Department of Endocrinology, L'institut du thorax, Nantes, France.; ^5^ Endocrine and Metabolic Consultants, Rockville, Maryland.; ^6^ Department of Pediatrics, Yale University, New Haven, Connecticut.; ^7^ Sanofi, Paris, France.; ^8^ Lexicon Pharmaceuticals, Inc., The Woodlands, Texas.; ^9^ Sanofi US, Inc., Bridgewater, New Jersey.; ^10^ Department of Medicine and Pediatrics, Barbara Davis Center for Diabetes, University of Colorado Denver, Aurora, Colorado.

**Keywords:** Sotagliflozin, Hypoglycemia, HbA1c, Efficacy beyond HbA1c, inTandem, SGLT1/SGLT2 inhibitors

## Abstract

***Background:*** Hypoglycemia rates usually increase when insulin treatment is intensified to improve glycemic control. We evaluated (post hoc) hypoglycemic rates in adult patients with type 1 diabetes (T1D) on sotagliflozin (a dual sodium-glucose cotransporter [SGLT] 1 and 2 inhibitor) in two phase 3, 52-week clinical trials (inTandem 1 and 2; NCT02384941 and NCT02421510).

***Materials and Methods:*** We analyzed rates of documented hypoglycemia (level 1, blood glucose ≥54 to <70 mg/dL) and clinically important hypoglycemia (level 2, glucose <54 mg/dL) in a patient-level pooled analysis (*n* = 1362) using a negative binomial model adjusted for hemoglobin A1c (HbA1c) at 52 weeks in patients receiving placebo, sotagliflozin 200 mg, and sotagliflozin 400 mg.

***Results:*** Rates of level 1 hypoglycemia events per patient-year were 58.25 (95% confidence interval: 50.26–67.50) with placebo, 44.86 (38.83–51.82; *P* = 0.0138 vs. placebo) with sotagliflozin 200 mg, and 45.68 (39.52–52.81; *P* = 0.0220) with sotagliflozin 400 mg. Sotagliflozin was also associated with lower rates of level 2 hypoglycemia: 15.95 (14.37–17.70), 11.51 (10.39–12.76; *P* < 0.0001), and 11.13 (10.03–12.35; *P* < 0.0001) for placebo and sotagliflozin 200 and 400 mg, respectively. The difference in rates of hypoglycemia with sotagliflozin versus placebo became more pronounced as HbA1c decreased.

***Conclusions:*** At week 52, level 1 and 2 hypoglycemia events were 22% to 30% less frequent with sotagliflozin added to optimized insulin therapy versus placebo in adults with T1D at any HbA1c level, with greater differences at lower HbA1c values. These findings support the use of sotagliflozin as an insulin adjunct in T1D.

## Introduction

As hemoglobin A1c (HbA1c) levels decrease, hypoglycemia risk increases for patients with diabetes who depend on insulin therapy.^1,2^ Nonsevere hypoglycemia—often a daily occurrence for many patients—impairs well-being and reduces productivity, and severe hypoglycemia may result in seizures, coma, or death.^3–7^ In the international Hypoglycemia Assessment Tool (HAT) study, which involved ∼8000 adults with type 1 diabetes (T1D), the estimated annual rate of any hypoglycemia was 73.3 episodes per patient-year. The rate of severe hypoglycemia was 4.9 events per patient-year, and up to 5% of HAT study participants reported being admitted to the hospital for hypoglycemia during the 4-week study period.^7^ Hypoglycemia places a substantial cost burden on the individual and society and poses a major barrier to achieving and maintaining optimal blood glucose levels.^6–8^ However, the increased risk of diabetes complications resulting from chronic hyperglycemia could also be considered a cost for patients who avoid intensifying insulin therapy for fear of hypoglycemia. Therefore, therapies are needed that improve overall glycemic control while reducing the risk of hypoglycemia.

Pramlintide, the only agent currently approved as an insulin-adjunct for the treatment of T1D in the United States, reduces HbA1c but is associated with an increased risk of severe hypoglycemia and gastrointestinal side effects.^9,10^ Recently, interest has focused on the potential of sodium-glucose cotransporter (SGLT) inhibitors in combination with insulin, as these agents have been shown to improve glucose control without increasing the risk of hypoglycemia or weight gain in patients with T1D.^11–18^ Sotagliflozin is a novel dual inhibitor of SGLT1 and SGLT2 that delays glucose absorption in the proximal gastrointestinal tract through SGLT1 inhibition, causing a blunting and reduction of postprandial glucose, and that decreases renal glucose reabsorption through SGLT2 inhibition.^19–21^ In the inTandem phase 3 program, consisting of one 24-week and two 52-week international, randomized, double-blind, placebo-controlled trials of sotagliflozin in combination with insulin in T1D, sotagliflozin significantly reduced HbA1c, fasting plasma glucose, body weight, and blood pressure (in patients with systolic blood pressure [SBP] ≥130 mmHg), and increased the proportion of patients achieving HbA1c <7.0%. Furthermore, decreases in documented hypoglycemia were observed in each inTandem trial.^11–14^

In the inTandem phase 3 trials, the rates of documented hypoglycemia were evaluated as plasma glucose ≤70 and ≤55 mg/dL.^11–13^ To further examine the incidence of hypoglycemia when sotagliflozin is used in combination with optimized insulin treatment, we conducted an exploratory post hoc analysis of pooled data from inTandem1 and 2 to compare the rate of hypoglycemia at each week 52 HbA1c value among patients receiving placebo and sotagliflozin over 1 year. For this post hoc analysis, we analyzed hypoglycemia incidence according to cutpoints recommended by the American Diabetes Association (ADA) and European Association for the Study of Diabetes (EASD), defined as level 1 (<70 mg/dL but ≥54 mg/dL) and level 2 hypoglycemia (<54 mg/dL).^22,23^

## Research Design and Methods

An exploratory post hoc analysis was conducted using pooled hypoglycemia and HbA1c data from week 52 of two phase 3, 52-week, multicenter, randomized, double-blind, placebo-controlled, parallel-group trials of oral sotagliflozin 200 or 400 mg once daily in combination with optimized insulin in adults with T1D who had inadequate glycemic control on insulin alone. The trials were conducted in the U.S. and Canada (inTandem1 [NCT02384941]) and Europe and Israel (inTandem2 [NCT02421510]). The Institutional Review Boards for each study center or the local Ethics Committees approved the protocol, consent form, and associated documents. All patients provided written informed consent. Trial designs have been previously described.^12,13^

### Study population

The inTandem program included men and nonpregnant women aged ≥18 years who had T1D treated with insulin delivered via multiple daily injections or continuous subcutaneous insulin infusion whose HbA1c was between 7.0% and 11.0% at screening. Full details have been previously reported.^12,13^ This pooled post hoc analysis included trial participants for whom an HbA1c value at week 52 was available.

### Interventions

Beginning 6 weeks before randomization, insulin therapy was optimized by adjusting basal and bolus doses to maintain fasting or preprandial blood glucose between 80 and 130 mg/dL and 1- to 2-h postprandial glucose ≤180 mg/dL based on self-monitored blood glucose. Patients were randomly assigned in a 1:1:1 ratio to placebo, sotagliflozin 200 mg, or sotagliflozin 400 mg, given as two tablets administered orally once daily. After randomization, participants entered a 24-week, double-blind core treatment period followed by a 28-week double-blind extension period. Insulin optimization continued throughout the 52-week trial for all patients, with guidance from an independent insulin dose monitoring committee from the start of the insulin optimization period through week 24 and investigator-led optimization thereafter.^12,13^

### Endpoints

The endpoints of this post hoc analysis included the number of level 1 hypoglycemia events per patient-year of exposure from baseline to endpoint (week 52), the number of level 2 hypoglycemia events per patient-year of exposure from baseline to week 52, and the incidence of positively adjudicated severe (level 3) hypoglycemia from baseline to week 52. Symptomatic and asymptomatic events were counted and included together in the analysis. In the original study protocols, level 1 hypoglycemia was defined as plasma glucose ≤70 mg/dL and level 2 as plasma glucose ≤55 mg/dL. To be consistent with ADA and EASD criteria,^22,23^ level 1 and 2 hypoglycemia were defined more strictly for this post hoc analysis: level 1, plasma glucose <70 mg/dL but ≥54 mg/dL; level 2, plasma glucose <54 mg/dL; and level 3 (severe), hypoglycemia was defined as any event that required assistance from another person or during which the patient lost consciousness or had a seizure (same definition used in the original inTandem studies).^12,13^

### Statistical methods

The estimated number of treatment emergent, documented hypoglycemia events per patient-year of exposure from baseline to endpoint was adjusted based on HbA1c at week 52, and 95% confidence intervals (CI) were determined. All symptomatic and asymptomatic hypoglycemic events were counted that occurred between day 1 and the end of treatment for which a documented blood glucose value was ≥54 and <70 mg/dL (level 1) or <54 mg/dL (level 2). The annualized rates were estimated by dividing the total number of events by the total exposure in years using a negative binomial model, with the total number of events per patient occurring from baseline to endpoint as the response variable, treatment and week 52 HbA1c as covariates, and log-transformed period duration (period defined as from baseline to endpoint) as an offset variable. As this is a post hoc analysis, *P* values were used for exploratory purposes only.

## Results

The pooled analysis from inTandem 1 and 2 trials included 448 patients receiving placebo, 460 receiving sotagliflozin 200 mg, and 454 receiving sotagliflozin 400 mg. Table 1 lists baseline demographics.

**Table 1.** Baseline Demographics of the Exploratory Analysis Population (Patients with a Hemoglobin A1c Value at Week 52)

	*Placebo (*n* = 448)*	*Sotagliflozin 200 mg (*n* = 460)*	*Sotagliflozin 400 mg (*n* = 454)*
Mean age ± SD, years	42.6 ± 13.3	44.8 ± 13.6	44.5 ± 13.0
Female sex, *n* (%)	207 (46.2)	224 (48.7)	227 (50.0)
Race, *n* (%)			
White	420 (93.8)	434 (94.3)	432 (95.2)
Black	8 (1.8)	9 (2.0)	6 (1.3)
Asian	4 (0.9)	7 (1.5)	4 (0.9)
Native Hawaiian or other Pacific Islander	2 (0.4)	2 (0.4)	0
American Indian or Alaska Native	0	1 (0.2)	0
Other	14 (3.1)	7 (1.5)	12 (2.6)
Mean diabetes duration ± SD, years	21.29 ± 12.04	21.95 ± 12.34	21.31 ± 12.16
Mean BMI ± SD, kg/m^2^	28.59 ± 5.28	28.88 ± 5.40	28.99 ± 5.11
Insulin therapy, *n* (%)			
CSII	190 (42.4)	195 (42.4)	190 (41.9)
MDI	258 (57.6)	265 (57.6)	264 (58.1)
Mean total daily insulin ± SD (IU/kg)	65.0 ± 36.4	62.8 ± 35.2	64.1 ± 34.3

BMI, body mass index; CSII, continuous subcutaneous insulin infusion; MDI, multiple daily insulin injections; SD, standard deviation.

At week 52, the mean HbA1c was 7.7% ± 0.9% (range 5.7%–11.9%), 7.4% ± 0.9% (5.6%–12.3%), and 7.3% ± 0.9% (5.5%–11.5%) in the placebo and sotagliflozin 200 and 400 mg groups, respectively.

**FIG. 1.**
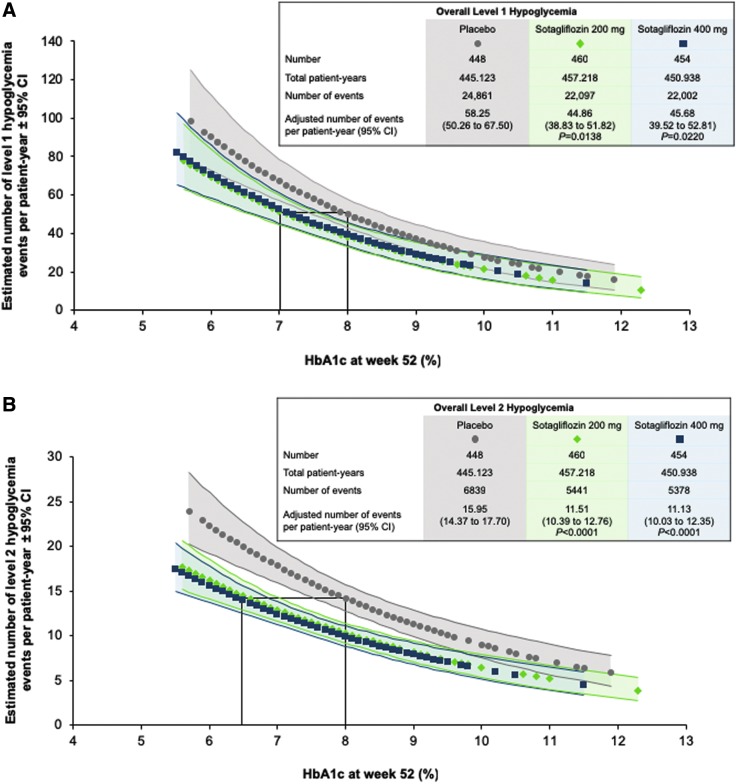
Estimated number of hypoglycemia events per patient-year as a function of HbA1c at week 52. **(A)** Level 1 hypoglycemia, defined as plasma glucose <70 mg/dL but ≥54 mg/dL. **(B)** Level 2 hypoglycemia, plasma glucose <54 mg/dL. Gray circles (placebo), green diamonds (sotagliflozin 200 mg), and blue squares (sotagliflozin 400 mg) indicate the rate of hypoglycemia at each HbA1c value, and the corresponding shaded areas depict the 95% CI. The box in each panel highlights the difference in HbA1c at equivalent rates of hypoglycemia. HbA1c, hemoglobin A1c; CI, confidence interval.

The adjusted number of events per patient-year of level 1 hypoglycemia was 58.25 (95% CI: 50.26–67.50) with placebo, 44.86 (38.83–51.82; *P* = 0.0138) with sotagliflozin 200 mg, and 45.68 (39.52–52.81; *P* = 0.0220) with sotagliflozin 400 mg. The rates of level 2 hypoglycemia were 15.95 (14.37–17.70) in the placebo group, 11.51 (10.39–12.76; *P* < 0.0001) in the sotagliflozin 200 mg group, and 11.13 (10.03–12.35; *P* < 0.0001) in the 400 mg dose group. The number of hypoglycemic events plotted versus HbA1c at week 52 (Fig. 1) was significantly reduced with sotagliflozin 200 and 400 compared with placebo (level 1, *P* < 0.05; level 2, *P* < 0.001).

Level 1 nocturnal hypoglycemia occurred at rates of 8.04 (95% CI: 6.97–9.27) events per patient-year in the placebo group, 6.30 (5.47–7.24; *P* = 0.0177 vs. placebo) with sotagliflozin 200 mg, and 6.25 (5.42–7.19; *P* = 0.0148) with sotagliflozin 400 mg. Rates of level 2 nocturnal hypoglycemia were 2.29 (2.00–2.64), 1.80 (1.56–2.06; *P* = 0.0149), and 1.70 (1.47–1.95; *P* = 0.0032) events per patient-year with placebo, sotagliflozin 200 mg, and sotagliflozin 400 mg, respectively.

Positively adjudicated severe (level 3) hypoglycemia occurred in 28 (6.3%) patients who received placebo and 12 (2.6%; *P* = 0.0091 vs. placebo) and 10 (2.2%; *P* = 0.0026 vs. placebo) patients receiving sotagliflozin 200 and 400 mg, respectively.

## Discussion

In this exploratory post hoc analysis of hypoglycemia as a function of HbA1c in patients with T1D receiving sotagliflozin or placebo in combination with optimized insulin therapy, level 1 hypoglycemia event rates were 22% lower with both doses of sotagliflozin compared to placebo, and level 2 was 28% and 30% lower with sotagliflozin 200 and 400 mg, respectively. After a year of treatment, mean HbA1c was 7.7%, 7.4%, and 7.3% with placebo and sotagliflozin 200 and 400 mg, respectively, and ranged from 5.5% to 12.3% across treatment groups. For any given HbA1c value, sotagliflozin 200 and 400 mg were associated with a substantially lower incidence of hypoglycemia than placebo. The proportions of patients reporting severe hypoglycemia were lower among those receiving sotagliflozin (2.6% and 2.2% with 200 and 400 mg, respectively) than placebo (6.3%).

Over 95% of study participants experienced at least one level 1 or level 2 event during the studies. With an incidence this high, data on rates (events per patient year), rather than incidence (proportions of patients affected), provide a more sensitive measure of risk. However, because the incidence of level 3 severe hypoglycemia (proportion of patients with at least 1 event) was much lower than that of level 1 and 2 hypoglycemia, and patients tended not to experience multiple episodes of the event, level 3 hypoglycemia is reported as incidence, as it is a more meaningful way to assess the impact of these events on patients.

The inverse relationship between HbA1c and hypoglycemia is a perennial challenge to optimal management of T1D. Patients who intensify insulin regimens to optimize glycemia and lower their risk of diabetic complications may experience more frequent hypoglycemic events. Hypoglycemia, and fear of it, negatively affects quality of life and ultimately undermines attempts to improve glycemic control.^7,24–26^ In the 52-week inTandem1 and 2 trials, sotagliflozin adjunct to insulin significantly decreased the incidence of clinically significant documented hypoglycemia (defined as plasma glucose ≤55 mg/dL in the main trials) relative to placebo, and positively adjudicated severe hypoglycemia occurred in 7.4%, 5.7%, and 4.4% of patients who received placebo, sotagliflozin 200 mg, and sotagliflozin 400 mg, respectively, in the pivotal trials. The use of sotagliflozin in combination with insulin also significantly reduced mean HbA1c, weight, insulin doses, and SBP (in patients with SBP ≥130 mmHg at baseline).^12,13^ As shown in a continuous glucose monitoring (CGM) substudy, sotagliflozin also reduced multiple measures of glycemic variability and significantly increased time in range, demonstrating reduced time in hyperglycemia without an increase in time <70 mg/dL.^27^ These changes were accompanied by significant improvements in treatment satisfaction and diabetes distress scales in the overall study populations.^12,13^

The present analysis supports these findings by demonstrating that sotagliflozin reduces the rate of ADA/EASD-defined level 1 (<70 mg/dL but ≥54) and level 2 (<54 mg/dL) hypoglycemia.^22,23^ The rates of level 1 and 2 hypoglycemia were similar with sotagliflozin 200 and 400 mg, but the proportion of patients reporting severe hypoglycemia was lowest in the 400 mg group.

Along with the improvements in glycemic variability observed in the inTandem CGM substudy, the reduction in hypoglycemia across the spectrum of HbA1c evident from the present analysis may at least partly account for the improvements in patient-reported outcomes seen in the main trials.^12,13,27^ In this study, patients receiving sotagliflozin who achieved an HbA1c of 7.0% experienced the same incidence of level 1 hypoglycemia (plasma glucose <70 mg/dL but ≥54 mg/dL) as patients who had an HbA1c of 8.0% while receiving insulin alone, and the same rate of clinically significant, level 2 hypoglycemia (plasma glucose <54 mg/dL) occurred with an HbA1c of 6.5% in the sotagliflozin group and 8.0% in the placebo group. Thus, patients whose HbA1c is lowered with the combination of sotagliflozin and insulin are less likely to experience hypoglycemia than those whose HbA1c reductions of a similar magnitude are achieved with insulin intensification alone.

In phase 3 studies with dapagliflozin in T1D (DEPICT trials), a final HbA1c of ∼8.0% was achieved after 24 weeks of treatment with dapagliflozin 10 mg, and the rates of documented and severe hypoglycemia were similar in the dapagliflozin and placebo groups.^16,17^ In phase 3 trials, empagliflozin 25 mg was associated with an HbA1c of ∼7.8% after 26 (EASE-3) and 52 (EASE-2) weeks of treatment. Investigator-reported documented hypoglycemia and severe hypoglycemia were similar between treatment groups, although patient diary reports of symptomatic hypoglycemia were lower with empagliflozin than with placebo.^18^ After 52 weeks, sotagliflozin 400 mg was associated with an HbA1c of 7.2% (inTandem1) and 7.4% (inTandem2), with lower rates of hypoglycemia compared to insulin alone.^12,13^ Overall, the results of these trials suggest that the lower the achieved HbA1c at the end of the trial, the higher the difference in rates of hypoglycemia, which tend to be lower in groups treated with the SGLT inhibitor.

Hypoglycemia has a greater impact on patients' day-to-day lives than hyperglycemic emergencies. Twice as many T1D Exchange participants reported an episode of severe hypoglycemia as diabetic ketoacidosis (DKA) over the previous 3 months.^28^ More emergency room visits are reported for severe hypoglycemia than DKA.^29^ Nevertheless, the risk of DKA is increased with SGLT inhibitors, including sotagliflozin.^30^ Sotagliflozin treatment was associated with an increased risk of DKA in the inTandem trials.^11–13^ Similar increases in DKA in phase 3 T1D trials were reported with the dosages of dapagliflozin and empagliflozin marketed for the treatment of type 2 diabetes.^16–18,31^ Sotagliflozin 200 and 400 mg and dapagliflozin 5 mg have been approved by the European Medicines Agency (EMA) as adjuncts to insulin in T1D.^32,33^ Both agents are still under review by the U.S. Food and Drug Administration (FDA). Clinicians using these medications in insulin-using patients should implement appropriate DKA risk-mitigation strategies as outlined in recent publications.^30,34^

A limitation of this study was that the relationship between hypoglycemia and insulin dose changes cannot be determined from the results presented here. In the year-long inTandem trials, total insulin doses at 52 weeks were 6% to 12% lower with sotagliflozin compared with placebo, with dose-dependent differences primarily driven by greater reductions in bolus insulin with sotagliflozin 400 mg.^12,13^

In conclusion, after 52 weeks, the addition of sotagliflozin to optimized insulin therapy was associated with a decrease in overall, nocturnal, and severe hypoglycemia across all HbA1c levels. Hypoglycemia increased as HbA1c decreased in all groups, but the sotagliflozin groups experienced a reduced rate of hypoglycemia relative to placebo at lower HbA1c levels. This approach could permit more patients to reach their target HbA1c without incurring an increased risk of hypoglycemia. Our data support the use of sotagliflozin in combination with insulin for the treatment of T1D.
